# Supplementary material for: The interactome of intact mitochondria by cross-linking mass spectrometry provides evidence for coexisting respiratory supercomplexes
Source: Mol Cell Proteomics. 2017 Dec 8;17(2):216–32. doi: 10.1074/mcp.RA117.000470 (PMC5795388; doi:10.1074/mcp.RA117.000470)
Supplement: Supplemental Data [file supp_RA117.000470_133922_0_supp_23978_fzffwf.zip › spectra_annotation/mito_spectra_annotation/1-1-1-1-2-1.pdf]

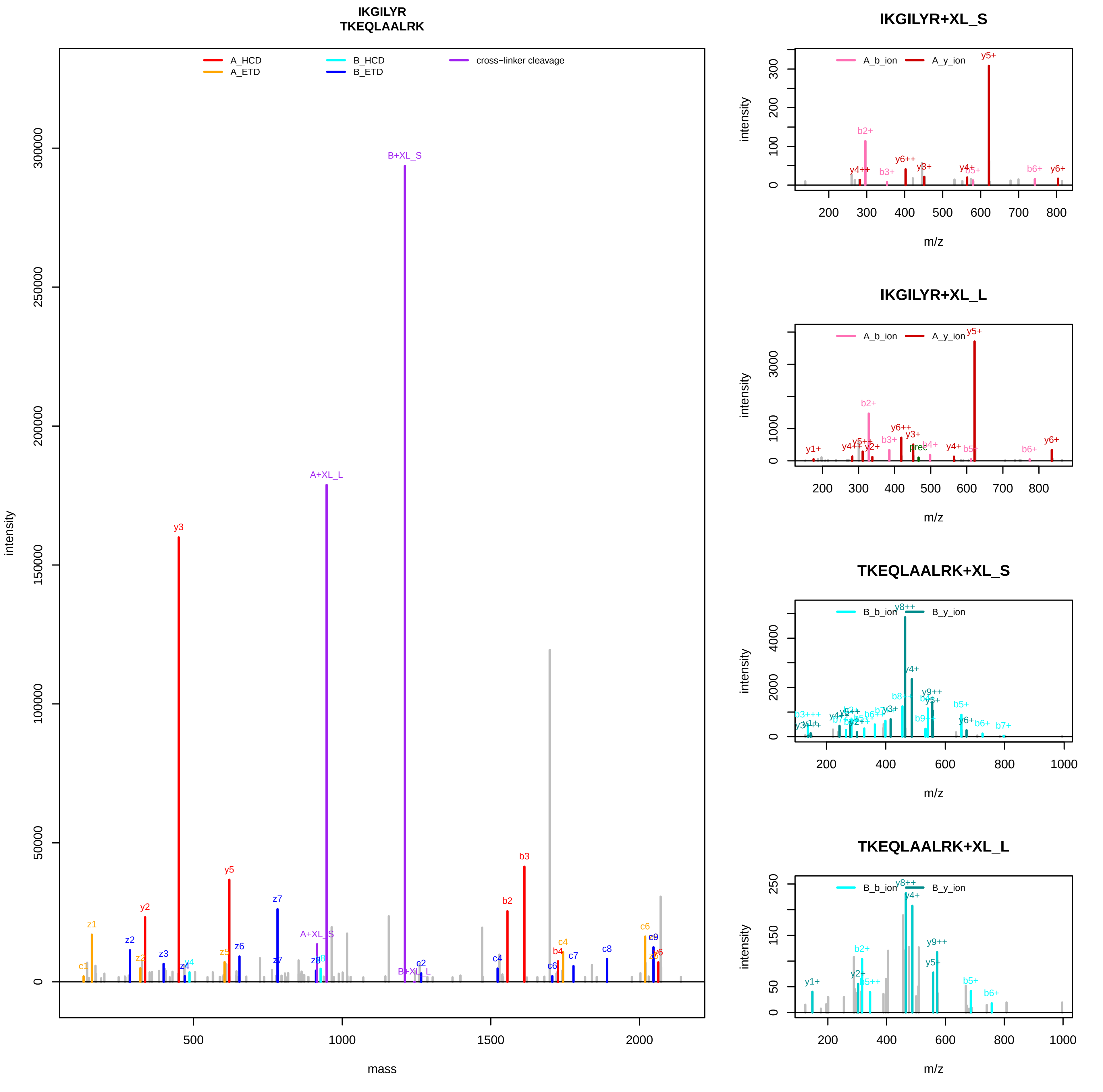
IKGILYR
TKEQLAALRK
A_HCD
A_ETD
B_HCD
B_ETD
cross−linker cleavage
intensity
mass
B+XL_S
A+XL_L
A+XL_S
B+XL_L
y3
y2
y5
z1
z2
z3
z4
z6
z7
c1
c2
c4
c6
c7
c8
c9
b2
b3
b4
IKGILYR+XL_S
IKGILYR+XL_L
TKEQLAALRK+XL_S
TKEQLAALRK+XL_L
A_b_ion
A_y_ion
B_b_ion
B_y_ion
m/z
y5+
b2+
y6++
y3+
y4++
b3+
y4+
b5+
b6+
y6+
y1+
y5++
y2+
prec
b4+
y8++
y4+
y9++
b5+
b7+
b6+
y1+
